# Mapping research evidence on quality of life in children with atopic dermatitis in sub-Saharan Africa: a scoping review protocol

**DOI:** 10.1186/s13643-022-02133-w

**Published:** 2022-11-23

**Authors:** Abraham Getachew Kelbore, Wendemagegn Enbiale, Anisa Mosam, Jacqueline M. van Wyk

**Affiliations:** 1 Department of Dermatology, College of Health Sciences and Medicine, Wolaita Sodo University, Wolaita Sodo, Ethiopia; 2 Department of Dermatology, Nelson R Mandela School of Medicine, University of KwaZulu-Natal, Durban, South Africa; 3 Department of Dermatology, College of Health Sciences and Medicine, Bahir Dar University, Bahir Dar, Ethiopia; 4 Department of Dermatology, Amsterdam Institute for Infection and Immunity (AII), Academic Medical Centre, Amsterdam UMC, University of Amsterdam, Amsterdam, Netherlands; 5 Nelson R. Mandela School of Medicine, University of KwaZulu-Natal, Durban, South Africa

**Keywords:** Eczema, Inflammatory skin diseases, Pediatric, Research evidence

## Abstract

**Background:**

Atopic dermatitis (AD) is a chronic, debilitating disease affecting children worldwide. Several studies have shown the disease to be a significant problem which leads to a diminished quality of life (QoL) for the affected children, but systematic evaluation of such studies in Africa is yet to be reported. Therefore, this scoping review aims to map research evidence on children with AD and their QoL in sub-Saharan Africa (SSA).

**Methods:**

The scoping review will follow the Arksey and O’Mally methodological framework. The electronic databases to be searched will include PubMed, EBSCOhost (Academic Search Complete, CINAHL, PsycINFO, and Health Sources), and Scopus and Google Scholar, for published literature between 2010 and 2021. The search strategy for the databases will include keywords, Medical Subject Headings terms, and Boolean operators. The reference list of the included sources of evidence and the WHO website will also be consulted for evidence relating to QoL of children with AD in SSA. Two independent reviewers will undertake abstract and full-text article screening with the guidance of eligibility criteria. This review will include studies conducted in SSA, and publications focusing on QoL and associated factors of AD in children. Data will be extracted from the included studies and analyzed qualitatively; NVIVO software V.11 will be used, and the emerging themes reported narratively. The mixed-method appraisal tool (MMAT) will be employed for quality appraisal of included studies.

**Discussion:**

We look forward to the findings of several studies that describe the QoL and associated factors among children with AD and that report on the use of different diagnostic criteria, severity scaling and QoL measuring scale tools used to ascertain the presence of AD, scale the severity of AD, and the impact of AD on QoL among children. This will help to improve clinical practice and the QoL of children with AD in SSA. The study findings will be disseminated through publication in a peer-reviewed journal, peer presentations, and presentations at relevant conferences.

**Conclusion:**

This study will add new knowledge on the QoL in children with AD in the SSA context. The study has the potential to inform research and clinical practice to impact the QoL of children with AD in SSA.

## Background

Atopic dermatitis (AD) is a chronic, intensely itchy, inflammatory skin disease that starts in childhood. It is a debilitating disease that affects up to 25% of children and 3% of adults, but varies significantly on a global scale [1, 2]. While the prevalence of AD in Africa and Middle Eastern countries has generally been lower than in Europe and North America, recent trends show an increasing prevalence of AD in low-income country contexts [3, 4]. Worldwide prevalence of AD for 6- to 7-year-old children was 14.2%. Also, the prevalence in the age group between 13 and 14 years was reportedly moderate in the eastern Mediterranean (10.8%) and Africa (15.2%) in comparison with the global prevalence of 12.8% for children in this age group [5]. The prevalence of AD among children in different African countries (Nigeria and Ethiopia 7.8% and 9.6% respectively) varies greatly [6, 7].

AD is a multifactorial disease, and studies suggest that the development of AD may be linked to genetic susceptibility, environmental, and lifestyle factors [8]. The main clinical features of AD include intractable itching and eczematous skin lesions that can be acute (erythema, vesicular eruption), sub-acute (ill-defined weepy crusted plaques), or chronic (well-defined dry lichenified plaques). Age of onset, xerosis, and family history of atopy are some of the essential features of AD [9]. AD is diagnosed based on clinical presentation using standardized diagnostic criteria in the absence of any definitive laboratory test to diagnose the condition [10, 11].

Treatment for AD includes frequent application of emollients, avoidance of irritating factors, education on the progress of the disease, and application of topical steroids (which can be complicated, uncomfortable, and stressful for children, their families, or caregivers). Not only does AD increase the financial burden on the family, but it is also closely associated with asthma and allergic rhinitis and results in significant morbidity, leading to school absenteeism and emotional stress in children [12]. Studies have reported how AD influences the QoL of children and their families or caregivers [12]. Sleep deprivation, irritability, anxiety, poor school performance, diminished self-esteem, and psychological disorders may be experienced by children with AD [13, 14]. As for their families or caregivers, studies have reported substantially greater levels of anxiety and depression due to sleep disturbance and absence from work [13].

Quality of Life is defined as “the individual’s perception of their position in life in the context of culture and value systems in which they live concerning their goals, expectations, standards, and concerns” [15]. Health-related quality of life (HRQoL) measures the QoL only in the context of the patient’s medical condition. In general, QoL comprises impact on living circumstances, family life, and school functioning [16]. Studies conducted in Western, European, and Asian countries confirm the correlation between the severity of AD with QoL of the affected child and their families [17–19]. There is, however, limited data on the QoL of children with AD in the African setting. It is therefore necessary to assess the QoL in children with AD and identify the factors that affect QoL, to improve treatment and understand the factors that negatively impact AD in SSA countries. Therefore, this scoping review aims to map research evidence on QoL and associated factors of AD in children in this region. It is anticipated that the review will reveal literature gaps and guide future research to inform policy decisions in SSA.

## Methods

This scoping review will constitute the first part of a large-scale study to investigate the QoL of AD-affected children in Ethiopia. The scoping review method will allow us to systematically map literature and describe existing research relating to AD in children in SSA. Aside from this, it will enable us to identify literature gaps and inform subsequent systematic review and primary study questions on AD in children. The scoping review will follow the steps outlined by Arksey and O’Malley [20] and Levac et al. [21], which included the following:

1. Identifying the research question
2. Identifying relevant studies
3. Study selection
4. Charting the data
5. Collating, summarizing, and reporting on the data.

### Identifying the research question

The main research question that this scoping review will address is: What research evidence exists on children (aged 0 to 15 years) with AD and their QoL within the last ten years in SSA?

The sub-review questions are as follows:

1. What research evidence exists on the QoL in children with AD?
2. What evidence is available on the associated/contributory factors that impact QoL of children with AD?
3. What instruments or assessment tools are available for assessing the QoL of children with AD?

### Eligibility criteria

Table 1 presents this review’s eligibility criteria as part of the Population, Concept, Context (PCC) framework defining the review questions.

**Table 1:** The PCC framework for defining eligibility of the scoping review questions

Criteria	Include	Exclude
Population	Children (aged from 0 to 15 years) with AD	Adolescents older than 15 years
Concepts	QoL (sleep disturbance, itching, mood change, emotional distress, irritability, poor self-esteem), associated or contributory factors, and QoL assessment tools/instruments	QoL of children living with HIV/AIDS, nutritional conditions, and chronic or acute illness that is not related to AD
Context	Sub-Saharan Africa (countries within the World Health Organisation Africa Region)	
Study design	Primary studies, systematic reviews, and QoL assessment tools	Abstracts without full-text, editorials, expert opinions/reviews
Time frame	2010 to 2021	
Publication language	English	

### Identifying relevant evidence sources

Searches will be conducted on PubMed, EBSCOhost (Academic Search Complete, CINAHL with full-text, PsycINFO, and Health Sources), Scopus, and Google Scholar from 2010 to 2021 for relevant published evidence sources to answer the review question. The search will employ keywords, Boolean terms (“AND” and “OR”), and medical subject headings to develop a comprehensive search strategy (Table 2). The syntax will be modified where needed. The team will also use the services of an experienced subject librarian to ensure that a robust review search strategy is followed. During the search, limitations on study design and language will be removed. The results of the search will be properly documented. Documentation will include the search date, database, keywords, search results, and number of articles that are eligible. To guide the electronic search strategy, the PRESS (Peer Review of Electronic Search Strategies) statement will be used. The reference list of the included sources of evidence and the WHO website will also be consulted for evidence relating to QoL of children with AD in SSA. To compile all relevant evidence sources and identify and remove duplicate records, we will use the EndNote X9 reference manager. AGK will search for the evidence sources assisted by the review team and import them into an EndNote X9 library created for this review.

**Table 2:** Pilot search to be conducted

Keywords searched	Date of search	Electronic database	Number of studies
(((("dermatitis, atopic"[MeSH Terms] OR "atopic dermatitis"[All Fields] OR "atopic eczema"[All Fields] OR "dermatitis, atopic"[MeSH Terms]) AND ("child" [MeSH Terms] OR "child"[All Fields] OR "children"[All Fields] OR "child s"[All Fields] OR "children s"[All Fields] OR "childrens"[All Fields] OR "childs"[All Fields])) OR ("paediatrics"[All Fields] OR "pediatrics"[MeSH Terms] OR "pediatrics"[All Fields] OR "paediatric"[All Fields] OR "pediatric"[All Fields]) OR ("paediatrics"[All Fields] OR "pediatrics"[MeSH Terms] OR "pediatrics"[All Fields] OR "paediatric"[All Fields] OR "pediatric"[All Fields]) OR ("adolescences"[All Fields] OR "adolescency"[All Fields] OR "adolescent"[MeSH Terms] OR "adolescent"[All Fields] OR "adolescence"[All Fields] OR "adolescents"[All Fields] OR "adolescent s"[All Fields])) AND ("africa south of the sahara"[MeSH Terms] OR "africa south of the sahara"[All Fields] OR "sub saharan africa"[All Fields] OR "sub saharan africa"[All Fields] OR "africa"[MeSH Terms] OR "africa"[All Fields] OR "Angola"[All Fields] OR "Benin"[All Fields] OR "Botswana"[All Fields] OR "Burkina Faso"[All Fields] OR "Burundi"[All Fields] OR "Cameroon"[All Fields] OR "Cape Verde"[All Fields] OR "Central African Republic"[All Fields] OR "Chad"[All Fields] OR "Comoros"[All Fields] OR "Congo"[All Fields] OR "Cote d'Ivoire"[All Fields] OR "Djibouti"[All Fields] OR "Equatorial Guinea"[All Fields] OR "Eritrea"[All Fields] OR "Ethiopia"[All Fields] OR "Gabon"[All Fields] OR "The Gambia"[All Fields] OR "Ghana"[All Fields] OR "Guinea"[All Fields] OR "Guinea-Bissau"[All Fields] OR "Kenya"[All Fields] OR "Lesotho"[All Fields] OR "Liberia"[All Fields] OR "Madagascar"[All Fields] OR "Malawi"[All Fields] OR "Mali"[All Fields] OR "Mauritania"[All Fields] OR "Mauritius"[All Fields] OR "Mozambique"[All Fields] OR "Namibia"[All Fields] OR "Niger"[All Fields] OR "Nigeria"[All Fields] OR "Reunion"[All Fields] OR "Rwanda"[All Fields] OR "Sao Tome and Principe"[All Fields] OR "Senegal"[All Fields] OR "Seychelles"[All Fields] OR "Sierra Leone"[All Fields] OR "Somalia"[All Fields] OR "South Africa"[All Fields] OR "Sudan"[All Fields] OR "Swaziland"[All Fields] OR "Tanzania"[All Fields] OR "Togo"[All Fields] OR "Uganda"[All Fields] OR "Western Sahara"[All Fields] OR "Zambia"[All Fields] OR "Zimbabwe"[All Fields])) AND ((clinical trial[Filter] OR comparative study[Filter] OR meta-analysis[Filter] OR observational study[Filter] OR randomized controlled trial[Filter] OR systematic review[Filter]) AND (humans[Filter]) AND (2010/1/1:2021/4/20[pdat]))	20/02/2021	PubMed	7394

### Selection of evidence source

Following the compilation of all evidence sources, the EndNote X9 library will be cleaned by deleting all duplicate records. All tools or forms that will be used for the selection of the evidence sources will be pilot tested, and the needed amendments made to ensure their accuracy and reliability. Subsequently, the EndNote X9 library will be shared among the review team. To reduce selection bias, two reviewers will conduct the title, abstract, and the full-text article screening independently. Guided by the scoping review eligibility criteria, the evidence sources will be sorted into either “include” or “exclude” groups by two independent reviewers. At the abstract stage, discrepancies that may arise will be discussed by the review team until a consensus is reached. A third reviewer will be included at the full-text screening stage. The University of KwaZulu-Natal library services will be used to retrieve all full-text articles with closed access publications. Also, we will send e-mails to the original authors for e-copies of relevant full-text articles. The various stages of the evidence sources selection will be appropriately documented using the PRISMA flow diagram [22].

### Charting the data

A data charting form will be developed and used to electronically capture relevant information from each of the included studies. This form will include the following: author and publication year, study title, aim of the study, study design, study setting, AD QoL measurement tool, AD severity scaling tools, AD diagnostic criteria, characteristics of study population, and other important findings like severity, sleep, social factors, and poor self-esteem that influence QoL of children with AD. The data extraction form will be piloted for consistency and reliability by two reviewers independently and amended if needed to ensure extraction of all relevant data.

### Collating, summarizing, and reporting the results

A narrative report will be produced to summarize the extracted data around the following outcomes: region of study, clinical severity and QoL measuring tools, and QoL of children with AD. Tables and graphs will be used to present the study characteristics if necessary. Qualitative thematic analyses will be used to collate, summarize, and report the results in the context of the overall study purpose by using NVIVO software V.11. The authors will read and reread the articles thoroughly, noting down the initial ideas to code. Coding interesting features of the data systematically across the entire articles and collating data relevant to each code will be done. We will develop the codes into potential themes. The description of the coding tree and thematic content analysis will be used to analyze the data. Data related to the QoL and AD severity scoring tools, factors related to the QoL, and QoL of children with AD will be extracted and coded. The analysis process will use the following steps:

1. Coding data from the selected articles
2. Categorizing the codes into themes
3. Displaying the data
4. Identifying key patterns in the data and identifying sub-themes
5. Summarizing and synthesizing

### Quality appraisal

The Mixed Method Appraisal Tool (MMAT) [23] will be adapted to assess the quality of the included primary studies. This scoping review will include qualitative, quantitative descriptive, and mixed methods study designs. The specific criteria to determine the appropriateness of each included study are outlined in Appendix.

Two reviewers (AGK and WE) will retrieve the data and assess each article for appropriateness to the study aims and relevance for inclusion based on selected review criteria. This will be verified by AM, who will produce a final list. The overall quality for each included study will be calculated according to the following MMAT guidelines (score = number of criteria met/total score in each domain). One point will be given for each question and a total score out of five will be calculated. This will be represented as a percentage that shows the quality of the included studies (Table 3), using the following descriptors.

**Table 3:** Quality scoring of studies

Verbal classification/rating	Description	Quality score
Very poor	Only one of the five criteria are met	20%
Poor quality	Only two of the stipulated criteria are met	40%
Fair quality	Three of the five criteria are met	60%
Good quality	Four of the five criteria are met	80%
Excellent quality	All five criteria are met	100%

## Discussion

Efficient treatment, QoL measurement in children with AD, and identification of factors that affect QoL are important for clinicians to understand that they are striving to reduce the negative impact of AD. Measuring the impact of skin disease on children is vital for clinical assessment, case management, follow-up, research, and audit reasons and can guide the management and inform decisions on resource allocation during policy development [24, 25]. QoL measures have been studied to evaluate outcomes for children with chronic health conditions. These are also a good index to monitor patient progress and response to provided treatment [26]. Parents of affected children report social difficulties and feelings of worry, guilt, blame, and frustration due to their child’s skin disorder [8, 27, 28]. In high-income countries, the QoL among children with AD has been well researched, whilst research in the context of African countries is limited. Studies, for example in South Africa and Uganda, have shown that AD has an impact on physical discomfort, low self-esteem, and emotional and social isolation for children and families [28, 29].

A scoping review of the evidence in the literature related to QoL of children with AD, factors that influence QoL, and QoL measurement tools, may help to inform future research to improve clinical practice and the QoL of children with AD in SSA.

## Data Availability

We have duly cited all studies, and data is presented in the form of references.

## Appendix

Selection of MMAT questions: specific criteria to determine the appropriateness for inclusion of each study.

Qualitative, quantitative descriptive and mixed methods study. The methodological quality criteria applied to evaluate qualitative studies included the following:

1. Is the qualitative approach appropriate to answer the research question?
2. Are the qualitative data collection methods adequate to address the research question?
3. Are the findings adequately derived from the data?
4. Do data sufficiently substantiate the interpretation of results?
5. Is there coherence between qualitative data sources, collection, analysis and interpretation?

The criteria to evaluate quantitative descriptive studies include the following:

1. Is the sampling strategy relevant to address the research question?
2. Is the sample representative of the target population?
3. Are the measurements appropriate?
4. Is the risk of non-response bias low?
5. Is the statistical analysis appropriate to answer the research question?

The criteria to evaluate mixed methods studies include the following:

1. Is there an adequate rationale for using a mixed-method design to address the research question?
2. Are the different components of the study effectively integrated to answer the research question?
3. Are the outputs of the integration of qualitative and quantitative components adequately interpreted?
4. Are divergences and inconsistencies between quantitative and qualitative results adequately addressed?
5. Do the different components of the study adhere to the quality criteria of each tradition of the methods involved?

## Abbreviations

AD: Atopic dermatitis
QoL: Quality of life
SSA: Sub-Saharan Africa
